# The comparison and use of tools for quantification of antimicrobial use in Indonesian broiler farms

**DOI:** 10.3389/fvets.2023.1092302

**Published:** 2023-03-10

**Authors:** Rianna Anwar Sani, Jaap A. Wagenaar, Tagrid E. H. A. Dinar, Sunandar Sunandar, Nofita Nurbiyanti, Imron Suandy, Gian Pertela, Elvina J. Jahja, Budi Purwanto, CORNERSTONE group, Tri S. Purwanto, Ingeborg M. van Geijlswijk, David C. Speksnijder

**Affiliations:** ^1^Division of Infectious Diseases and Immunology, Faculty of Veterinary Medicine, Utrecht University, Utrecht, Netherlands; ^2^Wageningen Bioveterinary Research, Lelystad, Netherlands; ^3^WHO Collaborating Center for Reference and Research on Campylobacter and Antimicrobial Resistance from a One Health Perspective/WOAH Reference Laboratory for Campylobacteriosis, Utrecht, Netherlands; ^4^Center for Indonesian Veterinary Analytical Studies (CIVAS), Bogor, Indonesia; ^5^Ministry of Agriculture of the Republic of Indonesia, Jakarta, Indonesia; ^6^Laboratory Services and Surveillance Department, PT Medion Farma Jaya, Bandung, Indonesia; ^7^Animal Health Department, PT Medion Farma Jaya, Bandung, Indonesia; ^8^Technical Education and Consultation Department, PT Medion Ardhika Bhakti, Bandung, Indonesia; ^9^Department of Population Health Sciences, Faculty of Veterinary Medicine, Utrecht University, Utrecht, Netherlands; ^10^University Farm Animal Clinic, Harmelen, Netherlands

**Keywords:** antimicrobial resistance, antimicrobial stewardship, veterinary antimicrobial use monitoring, poultry, Indonesia

## Abstract

**Introduction:**

Indonesia has a large broiler industry with extensive antimicrobial use (AMU) according to empirical evidence. However, there are no quantitative data of on-farm AMU. Quantification of AMU at farm level is crucial to guide interventions on antimicrobial stewardship (AMS). The objective of this study was to compare on-farm AMU monitoring methods, to assess which monitoring method is best suited to gain insight in the quantitative AMU at farm level in medium-scale Indonesian broiler farms.

**Method:**

AMU was calculated using four different indicators—mg/PCU (mass-based), TF_UDDindo_ (Treatment Frequency of Used Daily Dose, dose-based), TF_DDDvet_ (Treatment Frequency of Defined Daily Dose, dose-based), and TF_count − based_ (count-based)—for the total AMU of 98 production cycles with an average length of 30 days.

**Results:**

Broilers were exposed to an average of 10 days of antimicrobial treatments per production cycle, whereas 60.8% of the antimicrobials belonged to the Highest Priority Critically Important Antimicrobials (HPCIAs). For each pair of indicators, the Spearman rank correlation coefficient was calculated to assess if the production cycles were ranked consistently in increasing AMU across the different indicators. The correlation varied between 0.4 and 0.8.

**Discussion:**

This study illustrates the considerable difference in the ranking of AMU between the different indicators. In a setting comparable to medium-scale broiler farms in Indonesia, where resources are scarce and there is no professional oversight, the TF_count − based_ method is best suitable. Before implementing an AMU monitoring method, careful consideration of the use-indicators is paramount to achieve fair benchmarking.

## 1. Introduction

The increase of antimicrobial resistance (AMR) is seen as a major health threat for humans and animals worldwide. It is estimated that 1.27 million human deaths are attributable to bacterial AMR in 2019, and if no action is taken, AMR could become one of the biggest causes of human death by 2050 (1, 2). Multiple studies have illustrated that antimicrobial use (AMU) in livestock results in increased occurrence and dissemination of cross sectoral AMR. A reduction in AMU will reduce selection for AMR, which could eventually result in a decrease of AMR (3–7). A concern regarding AMR development in livestock is the frequent use of antimicrobials categorized by the World Health Organization (WHO) as Highest Priority Critically Important Antimicrobials (HPCIAs) for human medicine, such as 3^rd^ and 4^th^ generation cephalosporins, colistin and fluoroquinolones (8).

It is estimated that the majority of globally used antimicrobials (73%) are used in animals reared for food production, and the total amount used in animals is projected to increase by 11.5% by 2030, primarily in Asia (6, 9). This increase is most likely due to the intensification of the livestock industry to meet the growing demand for animal protein, particularly in Low- and Middle-Income Countries (LMICs) (5, 10). In many of these countries, professional veterinary oversight is lacking and antimicrobials can be purchased without a prescription, increasing the risk of the development of AMR due to indiscriminate use in livestock (11, 12).

With a population of 280 million people in 2022, Indonesia is the fourth highest populated country in the world. The Indonesian broiler sector accounts for 87% of the consumed meat, and empirical studies indicate that the broiler industry accounts for around 60% of the antimicrobial use in livestock (13, 14). Although a pilot surveillance study in 2019 has collected qualitative data on AMU, quantitative data on AMU at farm-level in the Indonesian broiler sector is lacking (15). There is no structural professional veterinary oversight over AMU (14). Availability of reliable AMU data at farm level is vital for antimicrobial stewardship (AMS) initiatives, targeting imprudent use, encouraging improvements in animal husbandry, biosecurity, and enabling detailed risk and trend analyses (16).

Setting up AMU monitoring systems involves various challenges, a major one being the choice of indicators for quantifying and reporting results. The indicator is a technical unit used to quantify an animal's exposure to antimicrobials. In the numerator, the indicator contains a unit of measurement (UM) that expresses the amount of antimicrobials used. Depending on the context and objective of the AMU monitoring system, a dose-based, mass-based or count-based UM can be used. A dose-based UM uses the number of standardized dosages (usually in mg/kg) in the numerator, a mass-based UM the total mass of the antimicrobials applied (usually in milligrams) in the numerator, and a count-based UM the number of administrations of an antimicrobial product. All UMs are applied during a defined period (e.g., production cycle, year). The denominator contains the animal population that is exposed to antimicrobials in a specific time period (16). By dividing the UM by the animal population that is exposed in the same time period, a treatment frequency (TF) can be calculated (quantity of AMU per time period). A major challenge is developing an AMU monitoring tool that is both easy to use in the local context and reliable in the collection, analysis and reporting of AMU data.

The objective of our study was to compare on-farm AMU monitoring methods for Indonesian broiler farms, to assess which monitoring method is best suited to gain insight in the quantitative AMU at farm level in medium-scale Indonesian broiler farms.

## 2. Materials and methods

Usage data from the CORNERSTONE project was used (17). This project is a longitudinal study which was initiated and coordinated by researchers from the Faculty of Veterinary Medicine of Utrecht University, in cooperation with the Center for Indonesian Veterinary Analytical Studies (CIVAS), Medion (Indonesian veterinary pharmaceutical company with direct relationships with poultry farmers) and FAO Indonesia, taking place from 2018 to 2023. In this project, a sample of nineteen medium scale broiler farms located in the western part of Java Island, Indonesia was selected for baseline data collection and an intervention study with the objective of increasing prudent AMU. The study is performed on medium-scale farms as this group forms the largest number of commercial farms in Indonesia. The farms were selected using a convenience sampling method from the client database provided by Medion and have either open- or semi-open housing systems. All farms were independent medium-scale commercial broiler farms with 5,000–20,000 broilers, utilizing developed housing and equipment, applying low to moderate biosecurity measures and usually marketing the birds commercially. During the recruitment process, farmers were explained the objective of the CORNERSTONE project was to gain insights in on-farm AMU in order to develop recommendations to optimize AMU. The implementation of these recommendations is voluntary, and farmers can quit the study at any point in time. All farmers signed an informed consent form prior to data collection. All traceable data was anonymized.

### 2.1. Selecting AMU indicators

Existing on-farm AMU monitoring systems were explored and the guideline “Quantification of veterinary antimicrobial usage at herd level and analysis, communication and benchmarking to improve responsible usage” (AACTING) was selected as the basis to develop an on-farm AMU monitoring system for medium-scale broiler farms in Indonesia (18). The different steps of the AACTING guideline were followed, which addresses the requirements for developing an AMU monitoring system regarding (1) data collection, (2) data analysis (including how to quantify AMU), (3) benchmarking, and (4) reporting (18).

In Step 2 (data analysis), the different options for unit of measurement (UM; the numerator of an indicator) to quantify AMU at farm level were considered. A UM of each of the three different categories—mass-based, dose-based, and count-based AMU metrics—was used for this study. Most farmers or farm managers on medium-scale poultry farms lack knowledge of prudent AMU and do not consult a veterinary professional when administering antimicrobials. This leads to a high variety of dosages. Using a standardized dose in the denominator on these farms could lead to an over- or underestimation of the actual AMU. The seriousness of this error was assessed by calculating the actual dose used. For each production cycle analyzed, we calculated two dose-based (Used Daily Dose (UDD) and Defined Daily Dose (DDDvet)), one mass-based (mg/kg) and one count-based (number of single-day treatments) UM. DDDvet as defined by the European Medicines Agency (EMA) uses a standardized dose derived from European data, whereas UDD is calculated using the measured use data from the studied farms (18). By calculating AMU both with UDD and DDDvet the applicability of the European standard DDDvet in the context of Indonesian medium-scale broiler farms is examined.

### 2.2. Data collection

AMU data was collected from at least four successive production cycles of one broiler house per participating farm. During each production cycle, an extension worker from CIVAS visited the farms three times (at the start, in the middle and just before harvest) to assemble and check the quality of the collected data. These extension workers instructed the farmers at the start of the project on what data they needed to collect. AMU data was collected using daily treatment records filled in by the farmers along with drug collection bins. The records contained the date and age of the broilers at application, the (brand) name of the veterinary medicinal product (VMP), purpose of use, the amount of the products used and the route of application. The drug collection bins were provided during the first visit from extension workers and emptied at the end of each cycle. The farmers were requested to place all used packages of administered products (except for feed packets) into the drug bins. During a cycle, the farmers were requested to send a picture or copy of the daily records every week. The farmers recorded the number of chicks at the start of the cycle, daily mortality rate, number of broilers sent to slaughter, and harvest weight. For some production cycles mortality rates were missing; in these cases, the number of broilers at the start of the cycle was used to calculate the denominator.

As the farmers did not record the average daily bodyweight of the broilers, the “standard” Indonesian growth curve for the Cobb strain was used to estimate the bodyweight of the broilers on each day of the production cycle (19). A standardized mean bodyweight throughout the cycle of 1.0 kg (as used in EMA guideline) was used for the mass-based indicator (20).

All collected data were entered and analyzed in Microsoft Excel 365 (Microsoft Corp., Redmond, USA). Quality check of the data was performed manually by checking the input. The exact (antimicrobial) contents of the VMPs that the farmers had applied were obtained through the Index for Veterinary Medicines Indonesia and cross-checked (Index list with used products) by an Indonesian veterinarian from CIVAS (21).

### 2.3. Calculation of the four different AMU monitoring tools

The first indicator calculated is mass-based and expressed in milligrams (mg) of active substance per Population Correction Unit (PCU). This indicator is calculated as (20):


$$\begin{array}{l} mass\text{-}based\;mg/PCU\\ =\frac{Total\;amount\;of\;active\;substance\;administered\;during\;a\;cycle\;\left(mg\right)}{standardized\;bodyweight\;\left(1.0\;kg\right){\;}^{*}N\;broilers\;\left(present\;at\;treatment\right)} \end{array}$$

The second indicator is the dose-based Treatment Frequency of used daily dose (TF_UDDindo_). The UM for this indicator was calculated for broilers specifically on the included production cycles of the study farms and was therefore named UDD_indo_. UDD_indo_ is defined as “the actual administered dose (as active substance in mg) per standardized bodyweight (kg) of an animal at treatment.” The UM UDD_indo_ needed to be established per treatment before the indicator TF_UDDindo_ can be calculated. UDD_indo_ was calculated per treatment as:


$$\begin{array}{l} dose\text{-}based\;UDD_{indo}\;\left(mg/kg\right)\\ =\;\frac{amount\;of\;active\;substance\;administered\;per\;treatment\;\left(mg\right)}{N\;treated{\;}^{*}standardized\;bodyweight\;at\;treatment\;\left(kg\right)} \end{array}$$

When the UDD_indo_ was calculated for each specific treatment during a cycle, the average UDD_indo_ for each active substance in all studied production cycles was calculated by dividing the sum of UDD_indo_ for a specific active substance by the number of treatments that contained the same antimicrobial active substance (Table 1).

**Table 1:** Overview of defined DDDvet and calculated UDDindo values used to calculate TF_DDDvet_ and TF_UDDindo_ respectively per production cycle.

**Antimicrobial group**	**Content**	**DDDvet mg/kg**	**UDD_indo_ calculated mg/kg**
Polymyxins (HPCIA)	Colistin	5.1	12.5
Fluoroquinolones (HPCIA)	Ciprofloxacin	Not available	30.0
Fluoroquinolones (HPCIA)	Enrofloxacin	10.0	31.4
Fluoroquinolones (HPCIA)	Flumequine	14.0	5.1
Macrolides (HPCIA)	Tylosin	81.0	20.3
Macrolides (HPCIA)	Erythromycin	20.0	19.3
Macrolides (HPCIA)	Spiramycin	73.0	7.8
Fosfomycin (CIA)	Fosfomycin	Not available	20.9
Aminoglycosides (CIA)	Spectinomycin	124.0	14.6
Aminoglycosides (CIA)	Neomycin	24.0	5.4
Penicillins (CIA)	Amoxicillin	16.0	23.1
Sulfonamides (HIA)	Sulfadiazine, trimethoprim	34.0	30.6
Sulfonamides (HIA)	Sulfaquinoxaline, natrium, pyrimethamin	60.0	16.5
Lincosamides (HIA)	Lincomycin	8.6	13.7
Tetracyclines (HIA)	Doxycycline	15.0	9.5
Tetracyclines (HIA)	Oxytetracycline	39.0	14.8

The Antimicrobial groups are: Highest Priority Critically Important Antimicrobials (HPCIA's), Critically Important Antimicrobials (CIA's) and Highly Important Antimicrobials (HIA's).

Once the UDD_indo_ for each active substance was determined, the TF_UDDindo_ was calculated by:


$$\begin{array}{l} dose\text{-}based\;TF_{UDDindo}\;\left(days\;of\;treatment/production\;cycle\;\left(30\;days\right)\right)\\ =\;\frac{\Sigma \;amount\;of\;active\;substance\;administered\;\left(mg\right)}{N\;treated{\;}^{*}standardized\;bodyweight\;at\;treatment\;{\left(kg\right)}^{*}UDDindo^{*}30\;days} \end{array}$$

The third indicator is comparable to TF_UDDindo_ but uses defined daily dosages instead of used daily dosages. This indicator is TF Defined Daily Dose (TF_DDDvet_). The DDDvet values were obtained according to the calculations by the European Medicines Agency (20). As the bodyweight plays a significant role in calculating AMU in broilers, the same standardized bodyweight at day of treatment was used as in TF_UDDindo_.


$$\begin{array}{l} Dose\text{-}based\;TF_{DDDvet}\;\left(days\;of\;treatment/production\;cycle\;\left(30\;days\right)\right)\\ =\;\frac{\Sigma \;amount\;of\;active\;substance\;administered\;\left(mg\right)\;}{N\;treated{\;}^{*}standardized\;bodyweight\;at\;treatment\;\left(kg\right){\;}^{*}DDDvet^{*}30\;days} \end{array}$$

The fourth indicator TF_count − based_ is count-based and expressed as the number of days under treatment per production cycle. If a VMP contained two antimicrobial active substances, it was counted as two separate treatments:


$$\begin{array}{l} Count\text{-}based\;TF_{count-based}\;\left(days\;of\;treatment/production\;cycle\;\left(30\;days\right)\right)\\ \;=\;\frac{n\;days\;of\;treatments\;of\;active\;substance\;per\;cycle\;}{30\;\left(average\;length\;of\;a\;production\;cycle\right)} \end{array}$$

The treatment frequencies therefore portray the proportion of days the broilers were under antimicrobial treatment during a standardized production cycle of 30 days.

### 2.4. Benchmarking and statistical analysis

An arbitrary benchmark analogous to the Dutch system was placed on the upper quartile in the ranking of each of the four indicators (22). The cycles within the highest AMU quartile (*n* = 25) were defined as “high AMU.”

For each of the four aforementioned indicators the AMU per production cycle was ordered from the lowest to the highest value. To test if these rankings for each specific indicator were correlated Spearman rank correlation coefficients (ρ) were calculated for each pair of indicators. The Spearman rank correlation coefficient measures the agreement between ranking methods and ranges from −1 (perfect negative agreement) to 0 (no agreement) to +1 (perfect positive agreement). The statistical significance test for a Spearman correlation assumes independent observations. The production cycles that were observed in this study were clustered in nineteen participating farms (four to six production cycles per farm). In the statistical analysis the intraclass correlation (ICC) was therefore calculated to check this assumption of independent observations. The Bonferoni adjusted *p*-value was calculated to compensate for the family wise error. For each pair of indicators, the number of production cycles ranked in the upper quartile for only one of the indicators but not for the other indicators was calculated. Additionally it was calculated how many cycles were ranked in the upper quartile in all four indicators.

## 3. Results

### 3.1. Application of the four different AMU monitoring tools

The checklist for each step provided by the AACTING guideline was filled out as part of collecting primary data for the context of the included medium scale broiler farms (Table 2).

**Table 2:** Checklist for developing an AMU monitoring system in the context of Indonesian medium-scale broiler farms.

**Requirements data collection according to AACTING guideline**	**Available options in the context of medium-scale broiler farms in Indonesia**
1. Identify Data sources	Data at farm level through:
	- Extension worker visits
	- Drug bins collecting all medicinal products used
	- Questionnaires filled in by farmers
2. Required information needed to calculate the quantity of each antimicrobial active substance used (numerator)	Use data required:
	- Unique ID/name of the antimicrobial containing VMP (through daily recordings)
	- Number of packages or amounts used (daily recordings)
	- Amount of active antimicrobial substances in all VMPs used [using Indonesian Index of Veterinary Medicine (IOHI) provided by ASOHI (association for Veterinary Medicine in Indonesia)]
	- Age at treatment (daily recordings)
3. Required information to calculate the size of population at risk of treatment (denominator)	- Flock size (recorded at farm level through daily recordings)
	- Flock size at time of treatment (recorded at farm level through daily recording)
	- Assumed biomass (bodyweight) per animal (standard weight set through Cobb growth curve provided by Medion)
4. Define data collection time windows as well as data lock points	- Window data collection: mean period of one production cycle of 30 days on medium-scale broiler farms in Indonesia
5. Determine how data should be provided	- Manual input in written daily recordings
6. Determine who should provide data	- Farmer (for this study with guidance of an extension worker)
7. Determine who can change the data	- Extension workers (validation of data input by farmer required)
	- System operators in case of errors
**Requirements Data Analysis according to AACTING guideline**	**Answers in context of medium-scale broiler farms in Indonesia**
1. Determine numerator for analysing the datas	Numerator for Mass-based (mg/PCU) and Dose-based (TF_UDDindo_, TF_DDDvet_) was expressed in milligrams of administered active substance. Numerator for Count-based indicator (TF_count − based_) was expressed in number of treatments (treatment being defined as a single-day treatment with one active substance)
2. Determine the denominator quantifying the size of population of animals at risk	Denominator for each indicator was expressed as:
	- Mass-based (mg/PCU): Standardized bodyweight (1.0 kg) multiplied by number of broilers present at treatment
	- Dose-based (TF_UDDindo_): standardized bodyweight (according to Cobb growth curve provided by Medion), multiplied by number of broilers present at treatment, multiplied by the calculated Used Daily Dose, multiplied by time period of 30 days
	- Dose-based (TF_DDDvet_): standardized bodyweight (according to Cobb growth curve provided by Medion), multiplied by number of broilers present at treatment, multiplied by the standardized Defined Daily Dose vet (calculated by EMA), multiplied by time period of 30 days
	- Count-based (TF_count − based_): time period of 30 (days)
3. Determine which AMU indicator best fits with the goal of the entire system and the AMU monitoring objectives	Based on
	- Data collection capacity without the aid of extension workers on the farms within this study
	- Objective of quantifying AMU at farm level and benchmarking in a fair manner
	The AMU indicator best suitable for these study farms would be Count-based (TF_count − based_)

Per farm, four to six production cycles were monitored (in total 98 production cycles across 19 farms), on average 5.2 per farm (Annex 1). In 97 production cycles, the broilers belonged to the Cobb strain, 1 production cycle used broilers from the Ross strain. Antimicrobials were used in 97 of the 98 (99%) production cycles. In total, 150 different VMPs were used, 53 of which contained antimicrobials. The daily recording forms were primarily used to analyze AMU per production cycle. The packages collected in drug collection bins were counted to cross-check the daily recording forms. All daily recording forms corresponded with the collected packages. The antimicrobials used belong to nine different antimicrobial classes, three of which are classified by the WHO as HPCIAs, three as Critically Important Antimicrobials (CIAs) and three as Highly Important Antimicrobials (HIAs) (23). Twenty-three VMPs contained a combination of two different antimicrobial substances.

The mean number of broilers that were present in the included study houses during a production cycle was 9,442 (ranging from 1,715–27,500, SD: 6,905). All four indicators ranked Cycle 3 on Farm 1 to have the highest AMU per standardized cycle (of 30 days). Leaving out the single production cycle in which no antimicrobials were used, all four indicators also identified the same production cycle (Cycle 3 on Farm 2) as having the lowest AMU. The mean AMU per standardized production cycle (*n* = 98) expressed in a mass-based indicator was 46.9 mg/PCU (SD: 58.3 mg/PCU). For the dose-based indicators, the mean TF_UDDindo_ was 0.3 (SD: 0.3) and TF_DDDvet_ was 0.6 (SD: 0.6). The mean TF_count − based_ was 0.3 (SD 0.2).

**Figure 1:**
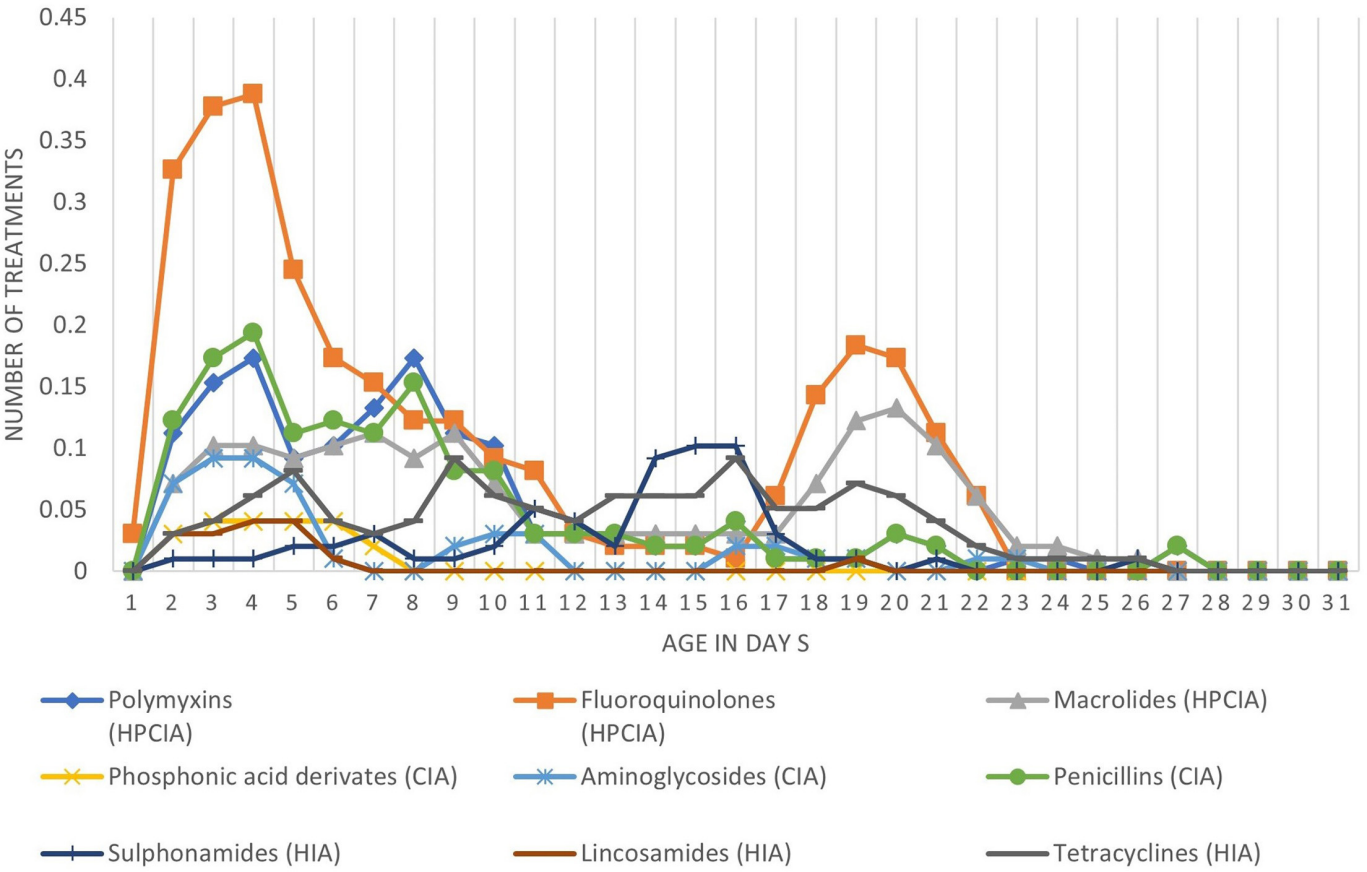
Average number of antimicrobial treatments per broiler per day of age divided in the different antimicrobial classes.

**Figure 2:**
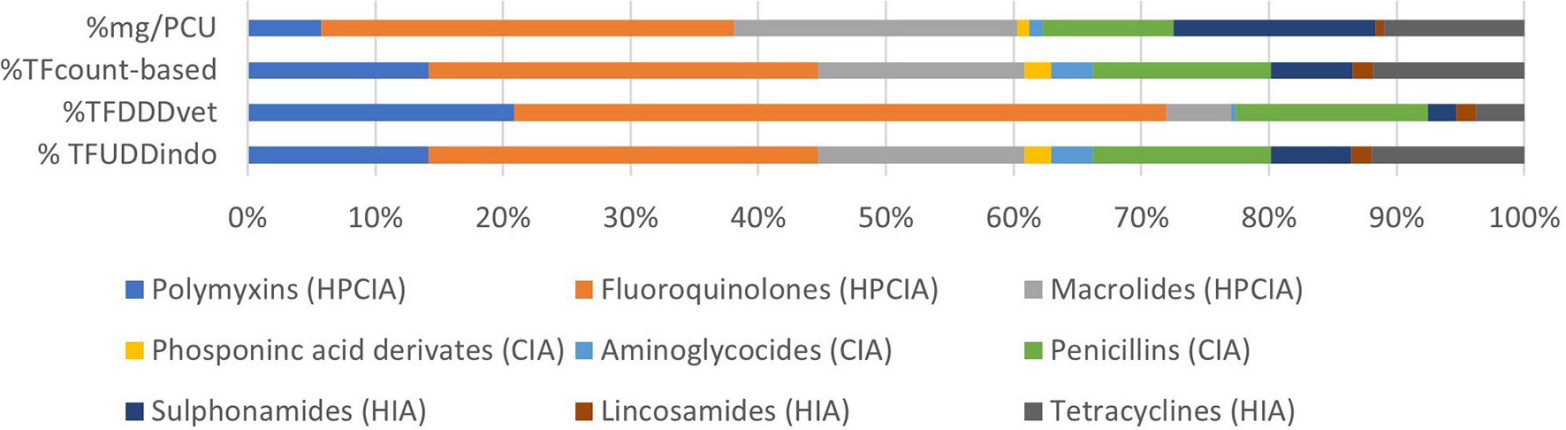
Proportion of antimicrobial classes used in all monitored cycles using the four different AMU indicators.

**Figure 3:**
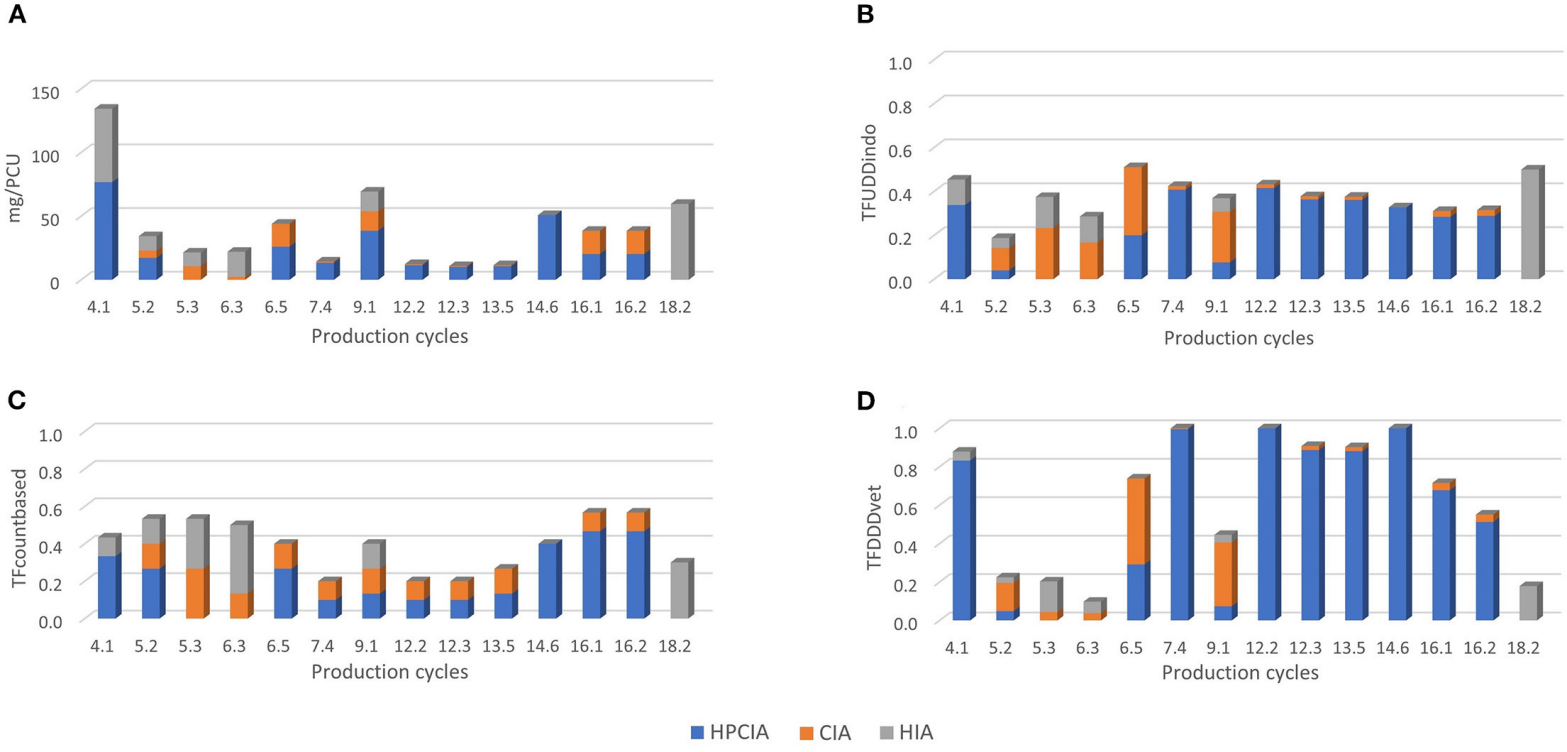
Distribution of AMU amongst the different priority antimicrobial classes as defined by WHO (HPCIA, CIA and HIA) of the 14 production cycles that were ranked as “high AMU” within only one indicator. Individual production cycles are labeled as [farm.cycle]; 4.1 means cycle 1 on farm 4. **(A)** Distribution of AMU defined as mg/PCU. **(B)** Distribution of AMU defined as TF_UDDindo_. **(C)** Distribution of AMU defined as TF_count − based_. **(D)** Distribution of AMU defined as TF_DDDvet_.

Figure 1 shows the number of treatments per antimicrobial class per day of age. On average, there were 10.2 antimicrobial treatment days per cycle. During the first 6 days of age, there is a high treatment incidence of fluoroquinolones (HPCIA) (e.g., in 39% of the monitored cycles, broilers were under fluoroquinolone treatment on Day 4 of the cycle), and a second period of high fluoroquinolone and macrolide (both HPCIA) treatment incidence from Days 17 to 23. Other antimicrobial classes show different dynamics of use during the first 23 days of the production cycle. Figure 2 shows the proportion of antimicrobial classes used in all monitored cycles using the different indicators. The proportions calculated as TF_count − based_, and TF_UDDindo_ show similar patterns, whereas the proportions for antimicrobial classes used calculated as mass-based (mg/PCU) and the TF_DDDvet_ indicator are different from the first two. Although overall TF_count − based_ and TF_UDDindo_ show similar patterns when calculated over all cycles, the variation becomes clear when individual cycles are analyzed (Figures 3A–D). For example, in Cycle 2 on Farm 12 (12.2) or Cycle 5 on Farm 13 (13.5), the proportion HPCIAs versus CIAs that were used differ considerably depending on whether TF_UDD − indo_ or TF_count − based_ was used.

Most AMU across all the monitored cycles belong to the HPCIAs, most of which were fluoroquinolones, irrespective of the indicator used (Figure 2). The percentage HPCIA use differs between indicators from 60.3 (mg/PCU) to 77.2% (TF_DDDvet_) (Figure 2). Depending on the indicator used, various production cycles were classified as “high AMU,” defined as having an AMU in the upper quartile within a specific indicator (Table 3).

**Table 3:** Pairwise comparison of AMU indicators using spearman rank correlation.

	**mg/PCU**	**TFUDD**	**TFDDDvet**	**Tfcount-based**
mg/PCU	1.00	0.78	0.69	0.69
	*N* = 0	*N* = 6	*N* = 8	*N* = 8
TFUDD		1.00	0.69	0.62
		*N* = 0	*N* = 8	*N* = 10
TFDDDvet			1.00	0.39
			*N* = 0	*N* = 15
TFcount-based				1.00
				*N* = 0

The values within the cell indicate the rho (ρ) coefficient and the number of farms ranked as “High AMU” [threshold upper quartile of AMU (N = 25)] with one indicator but below the threshold in the other indicatior in the pairwise comparison. The p-value for all Spearman rank correlation calculations was < 0.05. The color shades indicate the level of correlation found in this study: dark green represents perfect correlation and deep yellow represents no correlation.

The ICC was negligible (< 0.1) meaning that observations within each cluster were behaving as independent observations and the Spearman rank correlation test could be applied.

**Figure 4:**
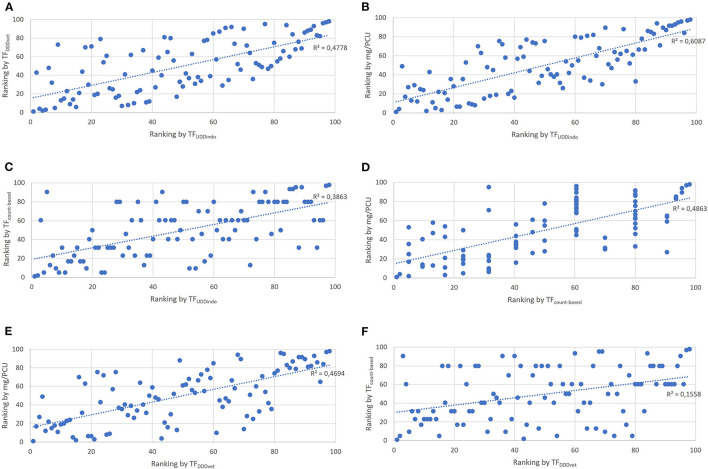
Scatter plots showing the correlation of individual production cycle AMU rankings between the 4 tested AMU indicators. **(A)** Correlation between TF_DDDvet_ and TF_UDDindo_. **(B)** Correlation between mg/PCU and TF_UDDindo_. **(C)** Correlation between TF_count − based_ and TF_UDDindo_. **(D)** Correlation between mg/PCU and TF_count − based_. **(E)** Correlation between mg/PCU and TF_DDDvet_. **(F)** Correlation between TF_count − based_ and TF_DDDvet_.

The lowest correlation found between two indicators was 0.4 (TF_DDDvet_ and TF_count − based_) and the highest correlation was 0.8 (mg/PCU and TF_UDDindo_) (Table 3, Figures 4A–F). The Bonferoni adjusted *p*-value for each of the six pairwise comparisons between indicators was < 0.05. Seven of the 25 production cycles in the upper quartile were classified as “High AMU” by all four indicators. Fourteen out of the 25 production cycles in the upper quartile were only marked as “High AMU” by just one indicator.

## 4. Discussion

In this study, we applied existing AMU indicators following the AACTING guidelines to gain insight into AMU at farm level on medium-scale broiler farms in Indonesia (18). Quantitative AMU data as well as data on the number of broilers present throughout the production cycle was used. Antimicrobials were used in 99% of the production cycles, although large variations between production cycles could be observed. Regardless of the unit of measurement (UM) used, the majority of antimicrobials used belonged to the HPCIA category. All UMs identified the same cycles as the cycle with the highest and lowest AMU, respectively. The UMs differed in the ranking of production cycles with increasing AMU. Nineteen production cycles were categorized as “high AMU” (upper quartile of AMU) for both the dose-Based UM TF_DDDvet_ and the mass-based UM TF_count − based_ together Only ten cycles were categorized as “high AMU” when calculated for both the mass-based UM mg/PCU and the dose-Based UM TF_UDDindo_ together.

### 4.1. Data collection and quality control

An effective monitoring system for AMU requires a measure of the amount of antimicrobials consumed and a measure of the population of animals at risk of treatment (16). Furthermore, ongoing systematic data should be collected to measure AMU change over time.

AMU data collection can be performed at different levels of aggregation or detail, and for different purposes. Indonesia reports national veterinary AMU at the level of species and administration route, and this is paralleled by collection of AMR data in poultry which is an ongoing surveillance system in Indonesia [personal communication Dr. Desmayanti; (24, 25)]. Data collection at the farm level, however, is important to understand why and how such large quantities of antimicrobials are used, identify high users, and provide the basis for developing AMS programs on farms (26). This study is the first that collected longitudinal and quantitative data on a sample of Indonesian broiler farms. This gave the opportunity to compare data-analysis systems for reporting and benchmarking, build experience in collecting data on broiler farms, and add quantitative data to the qualitative AMU studies that have been performed in the recent past in Indonesia. From our study, it is clear that an intensive follow-up is needed to collect reliable data from medium scale broiler farms in Indonesia. Even with the intensive follow-up there was no one guarantee that the AMU data was exact. Based on anecdotal reports from extension workers, farmers were not used to registering treatments precisely and appeared to find it difficult to make registration part of their daily routine. Intensive follow-up with frequent farm visits are a prerequisite for reliable data in situations where other data quality controls like intensive veterinary oversight are lacking. However, collecting on-farm data from a sample of farms as proxy for use, and extrapolate this to regional or even country level, will be a very time- and labor-consuming approach given the number of farms needed and the dispersed locations of farms (27). This is important to realize when deciding which AMU indicator will be used. A more detailed indicator (such as a dose-based indicator) where extensive data collection is required could be less suitable in this setting. Large-scale (>20,000 broilers) commercial farms, usually with developed housing and equipment, were not included in our study due to limited access to data (9). Due to a stricter farm management on large-scale farms, we can speculate that this might facilitate more thorough data collection. However, when data are collected to inform national policy, data from both large-scale and medium-scale farms should be included as there might be clear differences in usage. In the CORNERSTONE project, data collection is performed so that an intervention can be designed as part of an antimicrobial stewardship program.

### 4.2. Data analysis

Which UM is chosen often depends on the context (e.g., data availability, resources, objective of the monitoring system). A different choice of numerator (and thus indicator) can influence the interpretation of AMU at both national level (28, 29) and farm level (30, 31).

As antimicrobials classified as HPCIA are crucial in human medicine, it is paramount in AMU monitoring systems that the use of HPCIAs is not masked. When using a mass-based UM, the risk exists that the AMU can falsely appear to have decreased through switching to antimicrobial classes with a higher potency and so a lower required dose active substance, even though the duration of animal exposure to antimicrobials may not have changed. Remarkably, for the mass-based indicator mg/PCU in this study, the class of antimicrobials calculated to have been most used was the highly potent class of fluoroquinolones (HPCIA). This seemingly contradictory result can be explained by the three times higher dose of fluoroquinolones that was used on our study farms compared to the DDDvet, leading to a higher amount of milligrams being used than expected. This could also be the explanation for the (counter-intuitive) highest correlation between the UMs TF_UDDindo_ and mg/PCU.

A dose-based UM can be used to correct for the dosage. The challenge for dose-based UMs in a setting often lacking professional veterinary oversight, is that the recommended dose according to the SPC may not always reflect drug use in practice, as farmers frequently deviate from the recommended dosage (22, 26, 32). These variations were clear in this study, where the dosage of enrofloxacin used in the different cycles varied from 0.0017 to 203 mg/kg (the standardized dose according to EMA is 10 mg/kg). For fluoroquinolones this is due to huge variation in applied doses per farm. As a result of this variation in dosage per individual farm, even the standardized UDD_indo_, derived from the collected farm-level data leads to a varying over- or underestimation for each individual production cycle. This, in turn, leads to an incorrect ranking. Furthermore, comparing UDD_indo_ and DDD_vet_ shows that in this dataset the actual used dose (UDD_indo_) for colistin and enrofloxacin, both HPCIAs, was a 3-fold higher than the standardized DDD_vet_ as calculated by EMA (Table 1). In contrast, all other UDD_indo_ values were much lower than the DDD_vet_ values (Table 1). Considering the importance of HPCIA and the substantial difference in actual dose used and the DDDvet in this dataset, and varying under- and overestimation per individual farm by UDD_indo_, dose-based indicators have their restrictions in measuring AMU on medium-scale Indonesian broiler farms.

If a count-based UM is used, it is not necessary to have data available on the actual amount of antimicrobials used. Using a count-based UM thus requires less data, creating a lower burden for farmers to record data, but is less accurate compared to a dose-based UM if the goal is to examine the actual AMU at farm level. This is because it does not take into account the actual dosage applied, but counts every treatment with the same value (this value is “1”). However, the underestimation of use of potent antimicrobials, as would happen if a mass-based UM was used, is avoided when a count-based UM is used, because every treatment is weighted the same. However, it does not provide insight into under- or overdosing of a VMP, what appeared to happen frequently in our study population. It only counts the days that animals are under antimicrobial treatment in a predefined period, without weighing the quality of the AM treatment.

Besides choosing the numerator of the indicator for AMU, the AMU needs to be divided by a proxy for the targeted animal population to have comparable results (16). The weight of broilers increases by a factor of almost 40 (from 40 g to 1,500 g under Indonesian conditions) during their short life, which could imply a high risk of under- or overestimation of AMU when a single standardized animal weight is used (33). A mass-based UM for AMU usually uses slaughter weight, underestimating the effective exposure to antimicrobials per kilogram bodyweight, as most treatments often take place in the first week of production at low bodyweight. Due to varying management conditions of the farms in this study, there was also considerable variety in the actual bodyweight of the broilers at specific age on different farms, not always following the Cobb growth curve. A study by Kassabova in 2019 showed that using different weights to calculate dose-based AMU also significantly influences the outcome of the measurement (22). When available data on growth curves is limited, it could therefore be the best option to use a count-based UM, where the weight of the animals is not needed.

In summary, there are pros and cons for each UM for AMU. In the current setting of medium-scale broiler farms in Indonesia, the count-based UM seems most suitable (and realistic) to achieve a fair benchmarking of farms.

### 4.3. Benchmarking

Benchmarking AMU refers to comparing a farm's AMU with the AMU of the reference population (18). A prerequisite is that AMU for all entities in this population is quantified in a comparable manner. Using a different indicator can lead to a change in the way farms are ranked, which was clearly visible in this study (26, 34). Although some studies performed in broilers (34) and pigs (26) showed a correlation between the mass- and dose-based indicator, the correlation in this study was considerably lower [~0.6 (this study) compared to 0.8 (26)]. An explanation for this could be that the other studies were performed using data from countries where the administered dosages were more according to the SPC than in this study. A consistent over- or underestimation of the dosage would still result in a similar ranking of antimicrobial users, even though the exact values differ. However, if the over- or underestimation varies strongly, like in this study, the correlation automatically decreases.

Due to the limited number of participating farms and variation in the use of antimicrobials between production cycles, it was decided to benchmark per cycle instead of per farm in this study. This method is feasible in studies such as this one, where extensive supervision is possible. For this study, there was no preliminary data and benchmarking was only performed after data for all production cycles had been collected. Since farms have varying empty periods (in which no production cycle is running), quite some time can elapse between data collection of different cycles. For future studies, a timely benchmark is advised. This way, as soon as data is collected from one production cycle, it can immediately be reported back to the farmer whether or not their farm ranks as “high AMU.” Considering the duration of data collection, data analysis and efforts required to draft a report, benchmarking per farm is probably more realistic than per production cycle. Regardless of whether benchmarking is done within a smaller study or at a national level, similar farms should be used as a reference population. In this context, medium-scale farms should be compared with medium-scale farms and Large-scale farms with Large-scale farms.

### 4.4. Reporting

Reporting on the outcome of AMU quantifications is necessary for the improvement of AMS initiatives. Ongoing, systematic monitoring of on-farm AMU can guide targeted improvements of AMU as part of stewardship programs (16, 18). It is important to ensure the report is adjusted to the person it is addressed to (16). In our study, we reported back to farmers who mostly lack knowledge of prudent AMU and AMR. The report language should be understandable for this type of farmer and offer a clear overview of the AMU on their own farm compared to others within the reference population. In this study, practical suggestions on how to reduce AMU (particularly of HPCIAs) at farm level were added. If data is also reported to the government or at an international level, it is important to clearly define the reference population and add a time period to the AMU data (18). Anonymization of the participating farms is a prerequisite for each type of reporting and should be agreed upon when farms are included in studies.

Previous surveillance questionnaires concluded that AMU is widespread in the Indonesian broiler sector and that 80% is used preventively (15). Enrofloxacin is the most frequently used antimicrobial (15). With 10.2 average days under antimicrobial treatment per cycle and 82.6% of all treatments with the purpose of prevention or growth booster (2%) (data not shown), our results are comparable and there is an evident need to improve responsible antimicrobial use on medium scale broiler farms. An AMU monitoring system at farm level could be an effective tool to create insight for farmers in their use, which can in turn, assist in monitoring of the desired decreasing AMU.

## 5. Limitations of the study

Data were collected from only 19 farms, with close to 100 cycles. The cycles are not independent and might be clustered per farm for certain issues (e.g., dosing). The farms are selected by Medion based on their willingness to participate and are therefore not a representative sample of the AMU in medium-scale farms in their region. They might be more motivated to register treatments and open to advice. Due to the COVID-19 pandemic, data collection took longer than expected (it took place from 2019 to 2022). This might have influenced AMU habits, as during a 3-year time period, the antimicrobial treatment management could change.

## 6. Conclusion

Based on data from this study on 19 medium-scale broiler farms, the most feasible and fair method to benchmark medium scale farms is to use the UM TF_count − based_. One reason is that farmers from this sector are not yet used to extensive AMU data collection, as would be needed for the other indicators. Another important factor is the highly variable dosing practice found in this sector, which contrasts with the rigid legislation and veterinary oversight in European countries, for example. Consequently, a dose-based UM will not represent actual use and result in unfair benchmarking.

This study was the first to create insight into quantitative and qualitative AMU data at farm level in medium-scale broiler farms in Indonesia. The next step would be to use these tools on a larger sample of farms, and to use the outcomes for implementing interventions. Collecting AMU data at farm level in a database can subsequently help in monitoring AMU trends and aid policy makers in designing targeted AMS interventions. The easier count-based indicator TF_count − based_ would be best suitable for the current state of medium scale broiler farms in Indonesia. With this indicator the level of HPCIA use is not underestimated. Depending on future resources and possibilities to steer dosing practices, a dose-based indicator could be used as the successor of the count-based indicator.

## Data availability statement

The original contributions presented in the study are included in the article/Supplementary material, further inquiries can be directed to the corresponding author.

## Ethics statement

We conducted an observational study on the amount and type of antimicrobials used by farmers on regular commercial broiler farms in Indonesia. As we did not apply any intervention on humans nor animals, but only observed current antimicrobial use practices in commercial broiler farms based on written records and disposed drug packages, ethical review and approval was not required for the study. For the use and analysis of farm data (including antimicrobial use), we obtained written consent from the participating farmers. Furthermore, we anonymized all personal data so that no information can be traced back to individual farms.

## CORNERSTONE group

Tri S. Purwanto, Muhammad A. Bagaskara, Annisa Rachmawati, and Rangga Putra, Center for Indonesian Veterinary Analytical Studies (CIVAS), Bogor, Indonesia; Hannan Daradjat, Animal Health Department, PT Medion Farma Jaya, Bandung, Indonesia; Patricia Noreva, Ministry of Agriculture of the Republic of Indonesia, Jakarta, Indonesia; Riana A. Arief and Erianto Nugroho, Food and Agriculture Organization (FAO) Country Office of Indonesia, Jakarta, Indonesia.

## Author contributions

RA and TD coordinated data collection. The Cornerstone group under supervision of SS was responsible for the data collection and validation. RA and IG performed data analysis. RA prepared the manuscript. All authors reviewed the written manuscript and were involved in designing and conducting the study.
