# Corrigendum: The comparison and use of tools for quantification of antimicrobial use in Indonesian broiler farms

**DOI:** 10.3389/fvets.2025.1613626

**Published:** 2025-05-27

**Authors:** Rianna Anwar Sani, Jaap A. Wagenaar, Tagrid E. H. A. Dinar, Sunandar Sunandar, Nofita Nurbiyanti, Imron Suandy, Gian Pertela, Elvina J. Jahja, Budi Purwanto, CORNERSTONE group, Ingeborg M. van Geijlswijk, David C. Speksnijder

**Affiliations:** ^1^Division of Infectious Diseases and Immunology, Faculty of Veterinary Medicine, Utrecht University, Utrecht, Netherlands; ^2^Wageningen Bioveterinary Research, Lelystad, Netherlands; ^3^WHO Collaborating Center for Reference and Research on Campylobacter and Antimicrobial Resistance from a One Health Perspective/WOAH Reference Laboratory for Campylobacteriosis, Utrecht, Netherlands; ^4^Center for Indonesian Veterinary Analytical Studies (CIVAS), Bogor, Indonesia; ^5^Ministry of Agriculture of the Republic of Indonesia, Jakarta, Indonesia; ^6^Laboratory Services and Surveillance Department, PT Medion Farma Jaya, Bandung, Indonesia; ^7^Animal Health Department, PT Medion Farma Jaya, Bandung, Indonesia; ^8^Technical Education and Consultation Department, PT Medion Ardhika Bhakti, Bandung, Indonesia; ^9^Department of Population Health Sciences, Faculty of Veterinary Medicine, Utrecht University, Utrecht, Netherlands; ^10^University Farm Animal Clinic, Harmelen, Netherlands

**Keywords:** antimicrobial resistance, antimicrobial stewardship, veterinary antimicrobial use monitoring, poultry, Indonesia

In the published article, there was an error in Figure 1 as published. Due to some calculation- and classification errors in the raw data the average number of treatments per day of age had to be adjusted. The corrected Figure 1 and its caption appear below:

**Figure 1:**
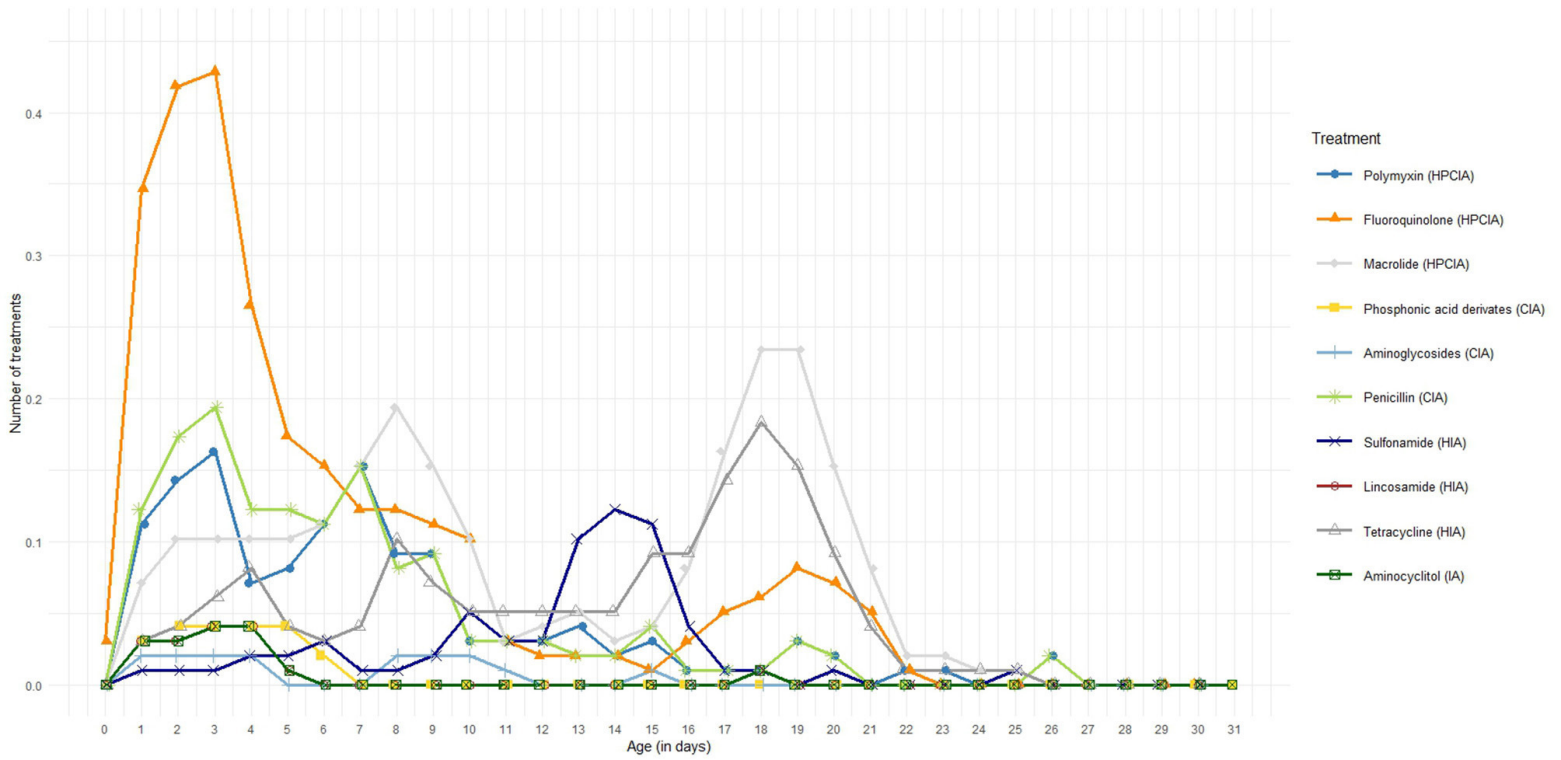
Average number of antimicrobial treatments per broiler per day of age divided in the different antimicrobial classes.

In the published article, there was an error in Figure 2 as published. Due to some calculation- and classification errors in the raw data the average number of treatments per day of age had to be adjusted. The corrected Figure 2 and its caption appear below:

**Figure 2:**
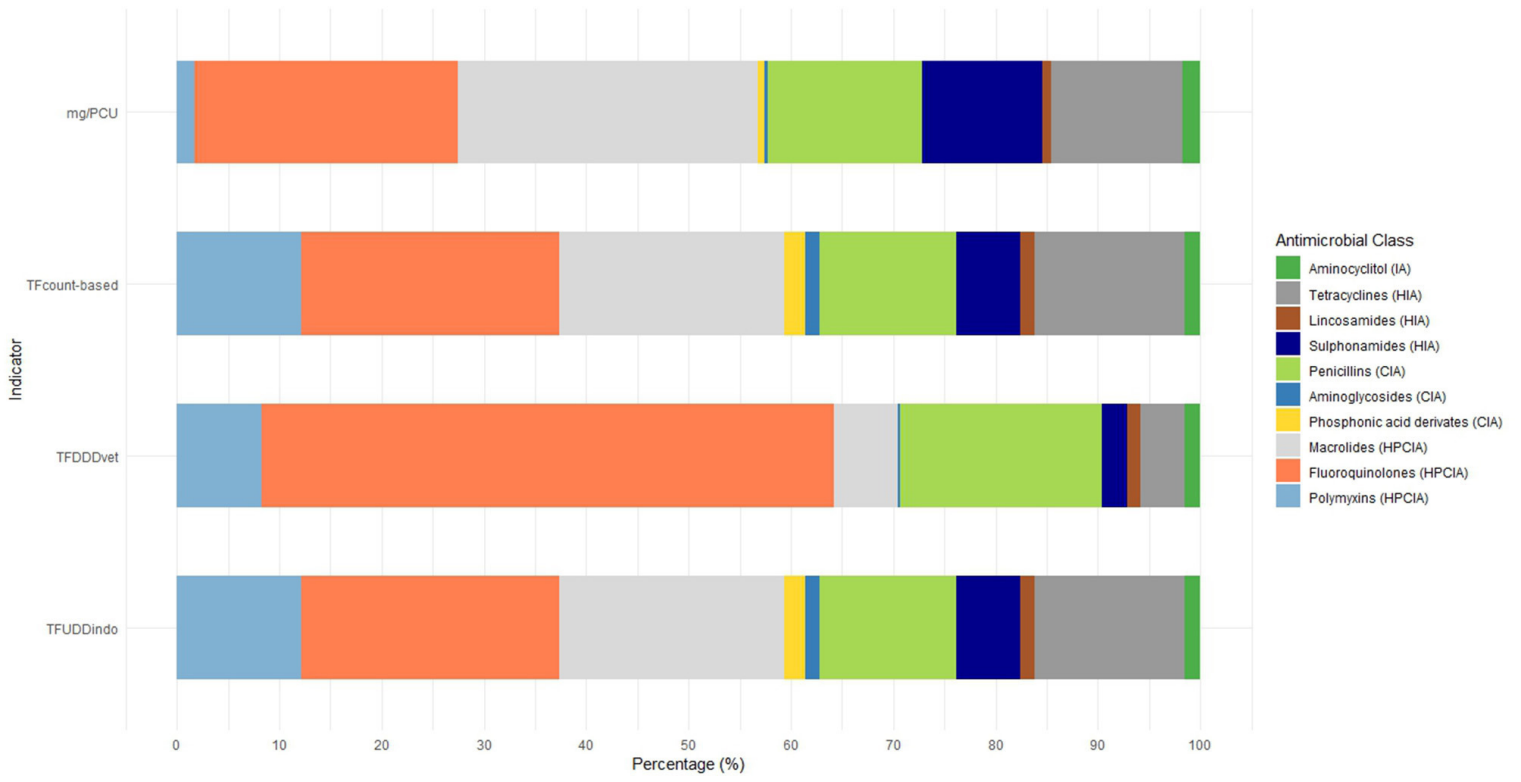
Proportion of antimicrobial classes used in all monitored cycles using the four different AMU indicators.

In the published article, there was an error in Figure 3 as published. Due to some calculation- and classification errors in the raw data the average number of treatments per day of age had to be adjusted. The corrected Figure 3 and its caption appear below:

**Figure 3:**
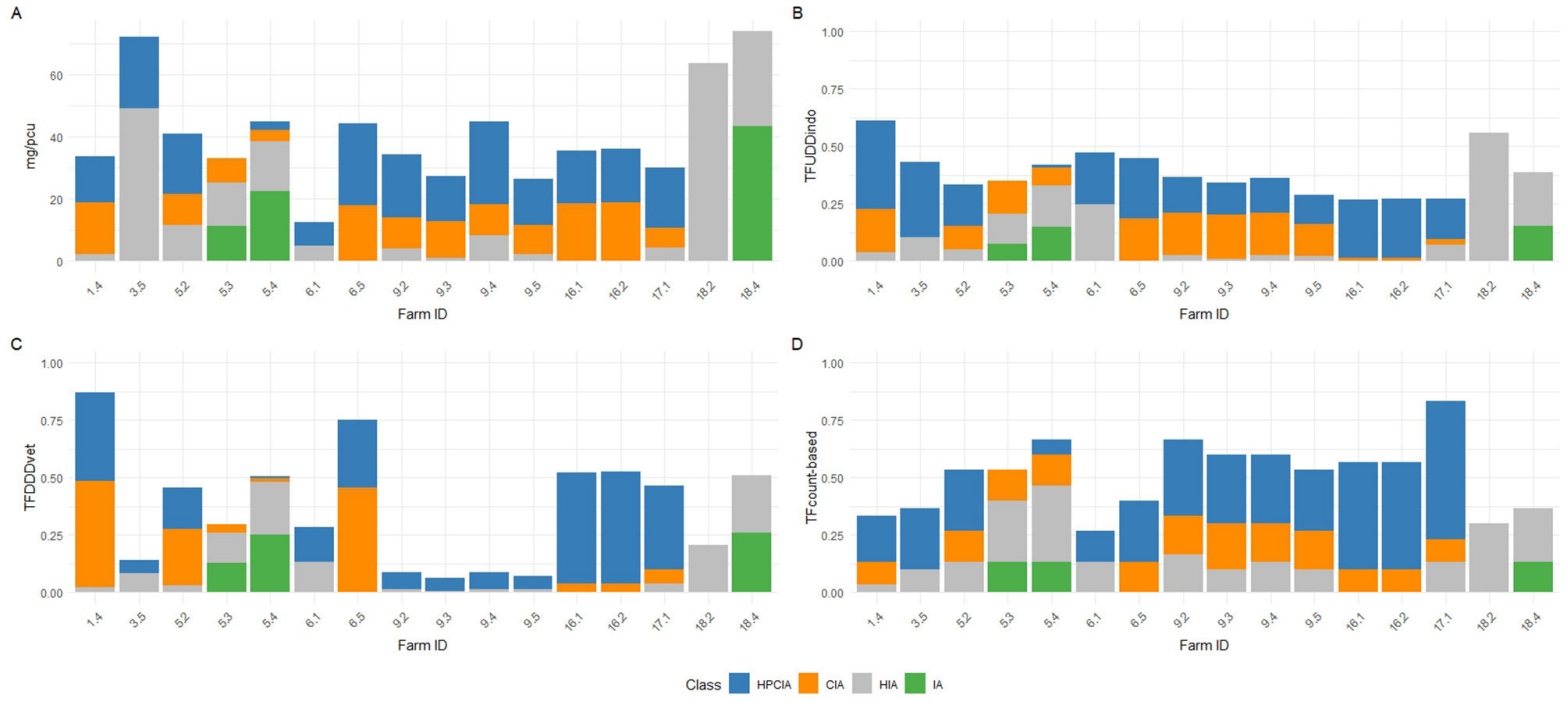
Distribution of AMU amongst the different priority antimicrobial classes as defined by WHO (HPCIA, CIA, HIA, and IA) of the 16 production cycles that were ranked as “high AMU” within only one indicator. Individual production cycles are labelled as [farm.cycle]; 1.4 means cycle 4 on farm 1. **(A)** Distribution of AMU defined as mg/PCU. **(B)** Distribution of AMU defined as TF_UDDindo_. **(C)** Distribution of AMU defined as TF_count−*based*_. **(D)** Distribution of AMU defined as TF_DDDvet_.

In the published article, there was an error in Figure 4 as published. Due to some calculation- and classification errors in the raw data the average number of treatments per day of age had to be adjusted. The corrected Figure 4 and its caption appear below:

**Figure 4:**
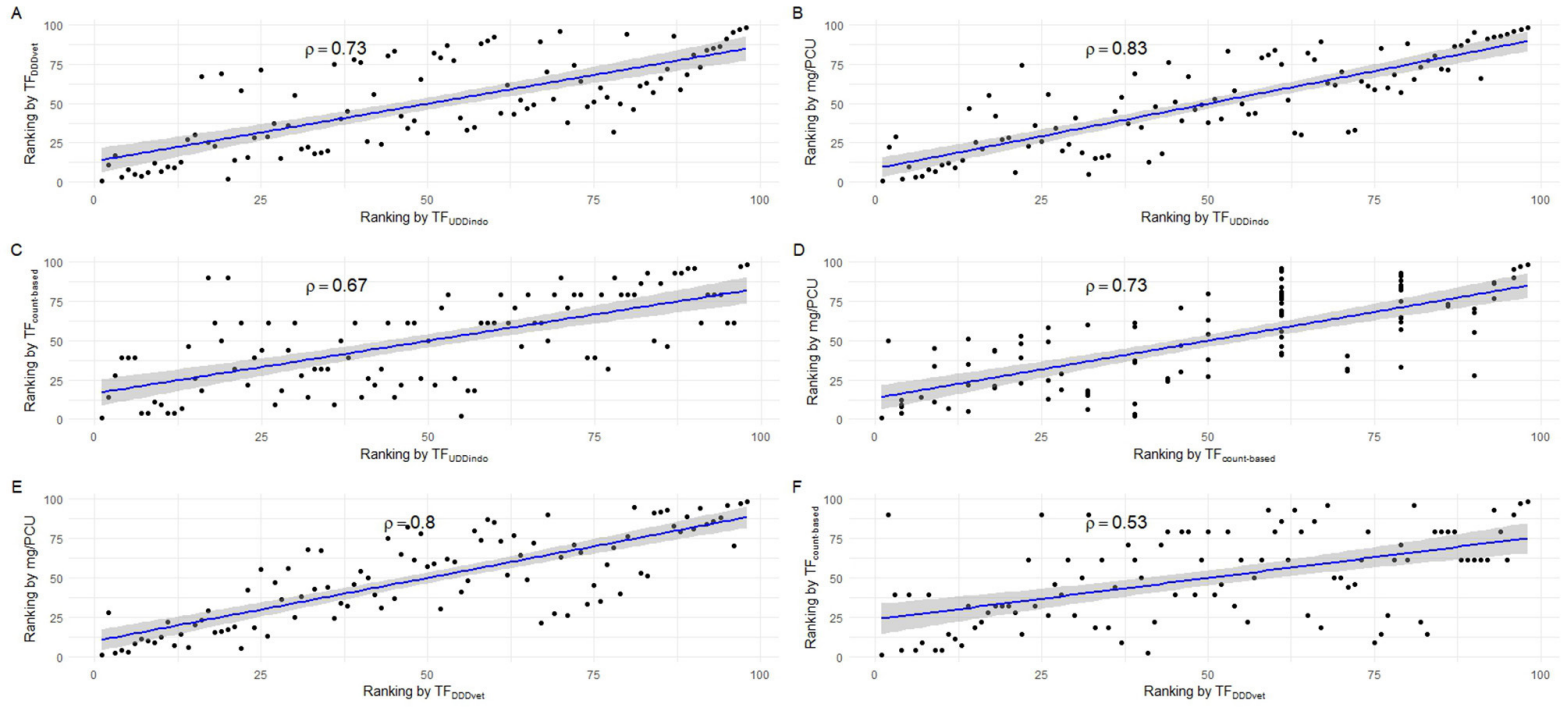
Scatter plots showing the correlation of individual production cycle AMU rankings between the 4 tested AMU indicators. **(A)** Correlation between TF_DDDvet_ and TF_UDDindo_. **(B)** Correlation between mg/PCU and TF_UDDindo_. **(C)** Correlation between TF_count−*based*_ and TF_UDDindo_. **(D)** Correlation between mg/PCU and TF_count−*based*_. **(E)** Correlation between mg/PCU and TF_DDDvet_. **(F)** Correlation between TF_count−*based*_ and TF_DDDvet_.

In the published article, there was an error in Table 1 as published. Due to some calculation- and classification errors in the raw data the average number of treatments per day of age had to be adjusted. The corrected Table 1 and its caption appear below:

**Table 1:** Overview of UDDindo values and DDDvet values.

**Antimicrobial ass**	**Antimicrobial**	**DDDvet (mg/kg)**	**UDD_indo_ (mg/kg)**
Polymyxins (HPCIA)	Colistin	5.1	5.7
Fluoroquinolones (HPCIA)	Ciprofloxacin	Not available	27.6
Fluoroquinolones (HPCIA)	Enrofloxacin	10.0	43.7
Fluoroquinolones (HPCIA)	Flumequine	14.0	5.2
Macrolides (HPCIA)	Tylosin	81.0	32.9
Macrolides (HPCIA)	Erythromycin	20.0	13.3
Macrolides (HPCIA)	Spiramycin	73.0	8.2
Fosfomycin (CIA)	Fosfomycin	Not available	21.5
Aminoglycosides (CIA)	Neomycin	24.0	5.7
Penicillins (CIA)	Amoxicillin	16.0	39.5
Sulfonamides (HIA)	Sulfadiazine (in combination with trimethoprim)	34.0	26.4
Sulfonamides (HIA)	Sulfaquinoxaline, natrium, pyrimethamin	60.0	13.5
Lincosamides (HIA)	Lincomycin (in combination with spectinomycin)	22.0	31.8
Tetracyclines (HIA)	Doxyxycline	15.0	8.2
Tetracyclines (HIA)	Oxytetracycline	39.0	16.0
Aminocyclitol (IA)	Spectinomycin (in combination with lincomycin)	38.0	63.7

The Antimicrobial groups are: Highest Priority Critically Important Antimicrobials (HPCIAs), Critically Important Antimicrobials (CIAs) and Highly Important Antimicrobials (HIAs). DDDvet values were obtained from the EMA (20). The UDD's were calculated as described in the material and method.

In the published article, there was an error in Table 3 as published. Due to some calculation- and classification errors in the raw data the average number of treatments per day of age had to be adjusted. The corrected Table 3 and its caption appear below:

**Table 3:** Pairwise comparison of AMU indicators using Spearman Rank Correlation; The values within the cell indicate the rho (ρ) coefficient and the number of farms ranked as “High AMU” [threshold upper quartile of AMU (N=25)] with one indicator but below the threshold in the other indicatior in the pairwise comparison.

	**mg/PCU**	**TF_UDDindo_**	**TF_DDDvet_**	**TF_count − based_**
mg/PCU	1.00	ρ = 0.83	ρ = 0.80	ρ = 0.73
	*N* = 0	*N* = 18	*N* = 8	*N*=20
TF_UDDindo_		1.00	ρ = 0.73	ρ = 0.67
		N = 0	*N* = 14	*N* = 26
TF_DDDvet_			1.00	ρ = 0.53
			N = 0	*N* = 26
TF_count − based_				1.00
				N = 0

The p-value for all Spearman rank correlation calculations was < 0.05.

In the published article, a correction has been made to **Abstract**, paragraph three. The incorrect sentence was written as: “Broilers were exposed to an average of 10 days of antimicrobial treatments per production cycle, whereas 60.8% of the antimicrobials belonged to the Highest Priority Critically Important Antimicrobials (HPCIAs).” This should have been written as “Broilers were exposed to an average of 11 days of antimicrobial treatments per production cycle, whereas 59.3% of the antimicrobials belonged to the Highest Priority Critically Important Antimicrobials (HPCIAs).”

In the published article, a correction has been made to **Abstract**, paragraph three. The incorrect sentence was written as “The correlation varied between 0.4 and 0.8.”. This should have been written as “The correlation varied between 0.5 and 0.8”.

In the published article, a correction has been made to **Results**, *Application of the four different AMU monitoring tools*, paragraph one. The incorrect sentence was written as “In total, 150 different VMPs were used, 53 of which contained antimicrobials.” This should have been written as “In total, 150 different VMPs were used, 41 of which contained antimicrobials.”

In the published article, a correction has been made to **Results**, *Application of the four different AMU monitoring tools*, paragraph one. The incorrect sentence was written as “The antimicrobials used belong to nine different antimicrobial classes, three of which are classified by the WHO as HPCIAs, three as Critically Important Antimicrobials (CIAs), and three as Highly Important Antimicrobials (HIAs). Twenty-three VMPs contained a combination of two different antimicrobial substances.” This should have been written as “The antimicrobials used belong to nine different antimicrobial classes, three of which are classified by the WHO as HPCIAs, two as Critically Important Antimicrobials (CIAs), three as Highly Important Antimicrobials (HIAs), and one as Important Antimicrobial (IA). Twenty-five VMPs contained a combination of two different antimicrobial substances.”

In the published article, a correction has been made to **Results**, *Application of the four different AMU monitoring tools*, paragraph two. The incorrect sentence was written as “The mean AMU per standardized production cycle (n=98) expressed in a mass-based indicator was 46.9 mg/PCU (SD: 58.3 mg/PCU). For the dose-based indicators, the mean TF_UDDindo_ was 0.3 (SD: 0.3) and TF_DDDvet_ was 0.6 (SD: 0.6). The mean TF_count − based_ was 0.3 (SD 0.2).” This should have been written as “The mean AMU per standardized production cycle (*n* = 98) expressed in a mass-based indicator was 58.5 mg/PCU (SD: 89.1 mg/PCU). For the dose-based indicators, the mean TF_UDDindo_ was 0.4 (SD: 0.4) and TF_DDDvet_ was 0.6 (SD: 0.7). The mean TF_count − based_ was 0.4 (SD 0.2).”

In the published article, a correction has been made to **Results**, *Application of the four different AMU monitoring tools*, paragraph three. The incorrect sentence was written as “On average, there were 10.2 antimicrobial treatment days per cycle. During the first six days of age, there is a high treatment incidence of fluoroquinolones (HPCIA) (e.g. in 39% of the monitored cycles, broilers were under fluoroquinolone treatment on Day 4 of the cycle), and a second period of high fluoroquinolone macrolide (HPCIA) and macrolide (both HPCIA) treatment incidence from Days 17 to 23.” This should have been written as “On average, there were 10.9 antimicrobial treatment days per cycle. During the first six days of age, there is a high treatment incidence of fluoroquinolones (HPCIA) (e.g. in 43% of the monitored cycles, broilers were under fluoroquinolone treatment on Day 3 of the cycle), and a second period of high macrolide (HPCIA) and tetracycline (HIA) treatment incidence from Days 17 to 23.”

In the published article, a correction has been made to **Results**, *Application of the four different AMU monitoring tools*, paragraph three. The incorrect sentence was written as “For example, in Cycle 2 on Farm 12 (12.2) or Cycle 5 on Farm 13 (13.5), the proportion HPCIAs versus CIAs that were used differ considerably depending on whether TF_UDD−*indo*_ or TF_count−*based*_ was used.” This should have been written as “For example, in Cycle 4 on Farm 5 (5.4) or Cycle 2 on Farm 9 (9.2), the proportion HPCIAs versus CIAs that were used differ considerably depending on whether TF_UDD−*indo*_ or TF_count−*based*_ was used.”

In the published article, a correction has been made to **Results**, *Application of the four different AMU monitoring tools*, paragraph four. This incorrect sentence was written as “The percentage HPCIA use differs between indicators from 60.3% (mg/PCU), to 77.2% (TF_DDDvet_) (Figure 2).” This should have been written as “The percentage HPCIA use differs between indicators from 56.7% (mg/PCU), to 70.5% (TF_DDDvet_) (Figure 2).”

In the published article, a correction has been made to **Results**, *Application of the four different AMU monitoring tools*, paragraph six. This incorrect sentences were written as “The lowest correlation found between two indicators was 0.4 (TF_DDDvet_ and TF_count−*based*_) and the highest correlation was 0.8 (mg/PCU and TF_UDDindo_) (Table 3, Figures 4A–F). The Bonferoni adjusted *p*-value for each of the six pairwise comparisons between indicators was < 0.05. Seven of the 25 production cycles in the upper quartile were classified as “High AMU” by all four indicators. Fourteen out of the 25 production cycles in the upper quartile were only marked as “High AMU” by just one indicator.” This should have been written as “The lowest correlation found between two indicators was 0.5 (TF_DDDvet_ and TF_count − based_) and the highest correlation was 0.8 (mg/PCU and TF_UDDindo_) (Table 3, Figures 4A–F). The Bonferoni adjusted *p*-value for each of the six pairwise comparisons between indicators was < 0.05. Ten of the 25 production cycles in the upper quartile were classified as “High AMU” by all four indicators. Sixteen out of the 25 production cycles in the upper quartile were only marked as “High AMU” by just one indicator.”

In the published article, a correction has been made to **Discussion**. The incorrect sentence was written as “Nineteen production cycles were categorized as “high AMU” (upper quartile of AMU) for both the dose-based UM TF_DDDvet_ and the mass-based UM _TFcount − based_ together. Only ten cycles were categorized as “high AMU” when calculated for both the mass-based UM mg/PCU and the dose-based UM TF_UDDindo_ together.” This should have been written as “Twelve production cycles were categorized as “high AMU” (upper quartile of AMU) for both the dose-based UM TF_DDDvet_ and the count-based UM TF_count − based_ together. Sixteen cycles were categorized as “high AMU” when calculated for both the mass-based UM mg/PCU and the dose-based UM TF_UDDindo_ together.”

In the published article, a correction has been made to **Discussion**, *Data analysis*, paragraph three. This incorrect sentence was written as “These variations were clear in this study, where the dosage of enrofloxacin used in the different cycles varied from 0.0017 to 203 mg/kg (the standardized dose according to EMA is 10 mg/kg).” This should have been written as “These variations were clear in this study, where the dosage of enrofloxacin used in the different cycles varied from 1.49 mg/kg to 273 mg/kg (the standardized dose according to EMA is 10 mg/kg).”

In the published article, a correction has been made to **Discussion**, *Data analysis*, paragraph three. This incorrect sentence was written as “Furthermore, comparing UDD_indo_ and DDD_vet_ shows that in this dataset the actual used dose (UDD_indo_) for colistin and enrofloxacin, both HPCIAs, was a 3-fold higher than the standardized DDD_vet_ as calculated by EMA (Table 1). In contrast, all other UDD_indo_ values were much lower than the DDD_vet_ values (Table 1).” This should have been written as “Furthermore, comparing UDDindo and DDDvet shows that in this dataset the actual used dose (UDDindo) for enrofloxacin, an HPCIA, was 3-fold higher than the standardized DDDvet as calculated by EMA (Table 1). In contrast, most other UDDindo values were much lower than the DDDvet values (Table 1).”

In the published article, a correction has been made to **Discussion**, *Benchmarking*, paragraph one. This incorrect sentences were written as “Although some studies performed in broilers (34) and pigs (26) showed a correlation between the mass- and dose-based indicator, the correlation in this study was considerably lower [~0.6 (this study) compared to 0.8 (26)]. An explanation for this could be that the other studies were performed using data from countries where the administered dosages were more according to the SPC than in this study. A consistent over- or underestimation of the dosage would still result in a similar ranking of antimicrobial users, even though the exact values differ. However, if the over- or underestimation varies strongly, like in this study, the correlation automatically decreases.” This should have been written as “Similar to some studies performed in broilers (34) and pigs (26) that showed a correlation between the mass- and dose-based indicator, the correlation in this study was comparable [−0.8]. However, greatly varying over- or underestimation of the dosage, like in this study, poses a risk of incorrect ranking.”

The authors apologize for these errors that occurred in the calculations, but state that this does not change the scientific conclusions and key messages of the article in any way. The original article has been updated.

