# Supplementary material for: The comparison and use of tools for quantification of antimicrobial use in Indonesian broiler farms
Source: Front Vet Sci. 2023 Mar 10;10:1092302. doi: 10.3389/fvets.2023.1092302 (PMC10036347; doi:10.3389/fvets.2023.1092302)
Supplement: Supplementary file 1 [file Table_1.DOCX]

***Annex***

Table 1 Amount of AMU per production cycle displayed using the four AMU indicators: mg/PCU (1), TF_UDD-indo_ (2),
TF_DDD-vet_(3), TFcountbased (4)

| Farm code | mg/PCU | TF_UDDindo_ | TF_DDDvet_ | TF_count-based_ |
| --- | --- | --- | --- | --- |
| 1.1 | 38.2 | 0.6 | 0.9 | 0.5 |
| 1.2 | 72.2 | 0.8 | 2.4 | 0.6 |
| 1.3 | 427.8 | 1.8 | 3.3 | 0.7 |
| 1.4 | 29.5 | 0.4 | 0.5 | 0.3 |
| 1.5 | 55.6 | 0.6 | 0.7 | 0.5 |
| 2.1 | 12.5 | 0.1 | 0.1 | 0.4 |
| 2.2 | 7.8 | 0.0 | 0.0 | 0.3 |
| 2.3 | 0.0 | 0.0 | 0.0 | 0.0 |
| 2.4 | 20.1 | 0.4 | 0.5 | 0.1 |
| 2.5 | 1.6 | 0.0 | 0.1 | 0.3 |
| 3.1 | 62.8 | 0.3 | 0.7 | 0.3 |
| 3.2 | 203.0 | 0.6 | 1.2 | 0.6 |
| 3.3 | 171.5 | 0.5 | 0.9 | 0.5 |
| 3.4 | 200.3 | 0.5 | 1.1 | 0.6 |
| 3.5 | 125.3 | 0.5 | 0.3 | 0.4 |
| 4.1 | 138.9 | 0.4 | 0.9 | 0.4 |
| 4.2 | 138.4 | 0.4 | 1.0 | 0.4 |
| 4.3 | 141.3 | 0.5 | 1.1 | 0.4 |
| 4.4 | 141.1 | 0.5 | 1.1 | 0.4 |
| 4.5 | 96.5 | 0.7 | 0.2 | 0.6 |
| 4.6 | 54.7 | 0.2 | 0.5 | 0.2 |
| 5.1 | 12.6 | 0.2 | 0.3 | 0.3 |
| 5.2 | 34.9 | 0.2 | 0.2 | 0.5 |
| 5.3 | 21.6 | 0.4 | 0.2 | 0.5 |
| 5.4 | 22.9 | 0.5 | 0.3 | 0.6 |
| 5.5 | 11.3 | 0.3 | 0.3 | 0.3 |
| 6.1 | 12.6 | 0.4 | 0.3 | 0.3 |
| 6.2 | 60.2 | 0.8 | 2.4 | 0.1 |
| 6.3 | 22.5 | 0.3 | 0.1 | 0.5 |
| 6.4 | 4.0 | 0.2 | 0.1 | 0.3 |
| 6.5 | 44.4 | 0.5 | 0.7 | 0.4 |
| 7.1 | 14.1 | 0.2 | 0.6 | 0.1 |
| 7.2 | 13.5 | 0.2 | 0.0 | 0.1 |
| 7.3 | 5.5 | 0.1 | 0.0 | 0.2 |
| 7.4 | 14.5 | 0.4 | 1.0 | 0.2 |
| 8.1 | 29.8 | 0.8 | 1.9 | 0.3 |
| 8.2 | 102.7 | 0.6 | 1.7 | 0.6 |
| 8.3 | 22.3 | 0.4 | 0.9 | 0.2 |
| 8.4 | 67.1 | 0.6 | 1.2 | 0.4 |
| 9.1 | 70.9 | 0.4 | 0.4 | 0.4 |
| 9.2 | 163.5 | 0.9 | 0.5 | 0.7 |
| 9.3 | 64.4 | 0.6 | 0.2 | 0.6 |
| 9.4 | 44.8 | 0.4 | 0.1 | 0.6 |
| 9.5 | 97.8 | 0.7 | 0.4 | 0.5 |
| 10.1 | 3.8 | 0.0 | 0.1 | 0.2 |
| 10.2 | 3.8 | 0.0 | 0.1 | 0.2 |
| 10.3 | 11.4 | 0.3 | 0.1 | 0.3 |
| 10.4 | 8.9 | 0.3 | 0.1 | 0.3 |
| 10.5 | 10.3 | 0.3 | 0.1 | 0.3 |
| 11.1 | 35.2 | 0.3 | 0.5 | 0.2 |
| 11.2 | 8.9 | 0.3 | 0.1 | 0.2 |
| 11.3 | 8.6 | 0.2 | 0.1 | 0.3 |
| 11.4 | 9.5 | 0.3 | 0.1 | 0.3 |
| 11.5 | 3.6 | 0.0 | 0.1 | 0.3 |
| 12.1 | 20.2 | 0.2 | 0.2 | 0.3 |
| 12.2 | 12.3 | 0.4 | 1.0 | 0.2 |
| 12.3 | 10.8 | 0.4 | 0.9 | 0.2 |
| 12.4 | 10.8 | 0.3 | 0.8 | 0.2 |
| 12.5 | 0.0 | 0.0 | 0.0 | 0.0 |
| 13.1 | 8.4 | 0.1 | 0.1 | 0.1 |
| 13.2 | 2.9 | 0.1 | 0.2 | 0.1 |
| 13.3 | 4.6 | 0.2 | 0.4 | 0.2 |
| 13.4 | 16.1 | 0.1 | 0.4 | 0.3 |
| 13.5 | 11.7 | 0.4 | 0.9 | 0.3 |
| 14.1 | 66.4 | 0.4 | 1.2 | 0.5 |
| 14.2 | 67.9 | 0.4 | 1.2 | 0.5 |
| 14.3 | 68.9 | 0.4 | 1.3 | 0.4 |
| 14.4 | 72.7 | 0.4 | 1.3 | 0.5 |
| 14.5 | 64.6 | 0.4 | 1.2 | 0.5 |
| 14.6 | 57.5 | 0.3 | 1.0 | 0.4 |
| 15.1 | 43.9 | 0.3 | 0.8 | 0.4 |
| 15.2 | 66.2 | 0.5 | 1.5 | 0.4 |
| 15.3 | 45.1 | 0.2 | 0.7 | 0.4 |
| 15.4 | 105.9 | 0.6 | 1.2 | 0.5 |
| 15.5 | 52.3 | 0.3 | 0.5 | 0.2 |
| 15.6 | 1.6 | 0.0 | 0.1 | 0.4 |
| 16.1 | 39.2 | 0.3 | 0.7 | 0.6 |
| 16.2 | 39.8 | 0.3 | 0.6 | 0.6 |
| 16.3 | 15.1 | 0.2 | 0.4 | 0.2 |
| 16.4 | 9.9 | 0.1 | 0.3 | 0.3 |
| 16.5 | 6.0 | 0.1 | 0.3 | 0.1 |
| 16.6 | 21.8 | 0.2 | 0.3 | 0.4 |
| 17.1 | 27.5 | 0.3 | 0.5 | 0.5 |
| 17.2 | 25.1 | 0.2 | 0.4 | 0.2 |
| 17.3 | 44.0 | 0.3 | 0.7 | 0.3 |
| 17.4 | 13.4 | 0.1 | 0.2 | 0.2 |
| 17.5 | 13.2 | 0.1 | 0.3 | 0.2 |
| 17.6 | 4.0 | 0.1 | 0.2 | 0.1 |
| 18.1 | 39.0 | 0.3 | 0.1 | 0.2 |
| 18.2 | 61.3 | 0.5 | 0.2 | 0.3 |
| 18.3 | 65.9 | 0.5 | 0.4 | 0.5 |
| 18.4 | 37.6 | 0.3 | 0.2 | 0.4 |
| 18.5 | 37.5 | 0.4 | 0.2 | 0.3 |
| 19.1 | 44.3 | 0.3 | 0.2 | 0.4 |
| 19.2 | 22.3 | 0.3 | 0.3 | 0.3 |
| 19.3 | 26.5 | 0.2 | 0.2 | 0.3 |
| 19.4 | 4.8 | 0.1 | 0.1 | 0.2 |
| 19.5 | 46.6 | 0.1 | 0.1 | 0.2 |
